# New strategies for sterilization and preservation of fresh fish skin grafts

**DOI:** 10.1038/s41598-024-51608-4

**Published:** 2024-01-13

**Authors:** Ahmed Ibrahim, Hossam M. Fahmy, Ghada Abd-Elmonsef Mahmoud, Mahmoud Soliman, Abdelnaby M. Elshahawy

**Affiliations:** 1 Veterinary Teaching Hospital, Faculty of Veterinary Medicine, Assiut University, Assiut, 71526 Egypt; 2 Laboratory and Transfusion Medicine, Faculty of Medicine, Ain Shams University, Cairo, Egypt; 3 Botany and Microbiology Department, Faculty of Science, Assiut University, Assiut, 71516 Egypt; 4 Department of Veterinary Pathology and Clinical Pathology, Faculty of Veterinary Medicine, Assiut University, Assiut, 71526 Egypt; 5 Department of Immunology, University of Texas Southwestern Medical Center, Dallas, TX 75235 USA; 6 Department of Physics, Faculty of Science, Assiut University, Assiut, 71516 Egypt

**Keywords:** Microbiology, Health care, Medical research

## Abstract

The introduction of fish skin as a biological dressing for treating burns and wounds holds great promise, offering an alternative to existing management strategies. However, the risk of disease transmission is a significant concern. Therefore, this study aimed to examine how established sterilization and preservation procedures affected fish skin grafts' microbiological and histological properties for long-term usage. Lyophilization of the fish skin graft followed by rehydration in normal saline for 15 min did not change the collagen content. Furthermore, gamma irradiation of the lyophilized fish skin graft at different lengths 5, 10, and 25 KGy showed a significant reduction in microbial growth (aerobic bacteria, aerobic yeasts, and fungi) at 15- and 30 days after the irradiation. However, exposure to 10 KGy was found to be the most effective intensity among the different gamma irradiation lengths since it preserved the collagen fiber content and intensity in the lyophilized fish skin grafts at 15- and 30 days after the irradiation. These findings provide efficient preservation and sterilization methods for long-term usage of the fresh Tilapia skin grafts used for biological dressings.

## Introduction

Fish skin has been used as a biological dressing in the management of burns^1–9^ and wounds^10^. It has also been used as a biological graft in neovaginoplasty in cases of vaginal agenesis^11,12^. The histological characteristics of fish skin and human skin are similar^13^. The fish skin contains a high amount of collagen, making it a suitable biomaterial for tissue engineering^9,13,14^.

Fish skin perishability still poses the most enormous preservation difficulties^15^. It must be kept chilled or frozen, and even then, it has a very short shelf life^16^. Several diverse damage mechanisms, including microbiological spoilage, autolytic degradation, and lipid oxidation, are responsible for the depreciating of fresh fish skin during storage^17^. Therefore, most clinical practices have used fish skin grafts in fresh form for wound and burn management^1,3,5–10^.

Lyophilization, known also as freeze drying or cryodesiccation, is a low-temperature dehydration process involving freezing the product, lowering the pressure, then removing the ice by sublimation. This is in contrast to dehydration by most conventional methods use heat to evaporate water^18^.

Lyophilization can be a potential method for the long-term storage of fish skin grafts. In a previous study, lyophilization was addressed as a method for preserving fish skin grafts^2^. However, applying several detergents and sterilizing agents (e.g., chlorhexidine) to fish skin before lyophilization raises concerns about the integrity of collagen and the targeted material in the fish skin^2,9,10,13^. Moreover, the histopathology and microbiology of these lyophilized fish skin grafts were not evaluated before being used clinically.

The microbial colonization of the wound is an essential factor that affects the healing process of wounds^19^. Different sterilization methods have been described for biological dressings. These include physical methods such as irradiation and chemical techniques including treatment with chlorhexidine, povidone iodine, ethylene oxide gas, silver nanoparticles, and ozone^10,13,20^. Gamma irradiation represents an effective sterilization method as it has direct and indirect effects on microbial DNA^21^. Different lengths of Gamma irradiation have been used to sterilize fish skin (25, 30, and 50 kGy) without reference to its impact on collagen content^1,2,13^ or antimicrobial efficiency^2,9^.

The current study’s objectives are (1): to describe the optimized method of Tilapia skin lyophilization and (2): to define the standard length of gamma irradiation for sterilization of lyophilized Tilapia skin with special consideration to collagen integrity and microbial count of fish skin.

## Materials and methods

### Ethics statement

The Research Ethics Committee (REC) of the Faculty of Veterinary Medicine, Assiut University, Assiut, Egypt, has approved all the procedures in this study in accordance with the Egyptian bylaws and OIE animal welfare standards for animal care and use in research and education. All methods were performed in accordance with relevant guidelines and regulations.

### Fish skin sampling

The fish skin was collected from fresh Nile tilapia *(Oreochromis niloticus)* (weigh: 620 ± 35 gm; standard length: 20 ± 3 cm), obtained from The Aquatic Medicine Unit, Faculty of Veterinary Medicine, Assiut University, Assiut, Egypt*.* Fish were euthanized physically by decapitation. After removal the fish scales, the skin was dissected from the underlying tissue, cut into strips (6 × 2 cm), and then washed in sterile normal saline. Lyophilization was performed on skin strips.

### Fish skin lyophilization

**Figure 1:**
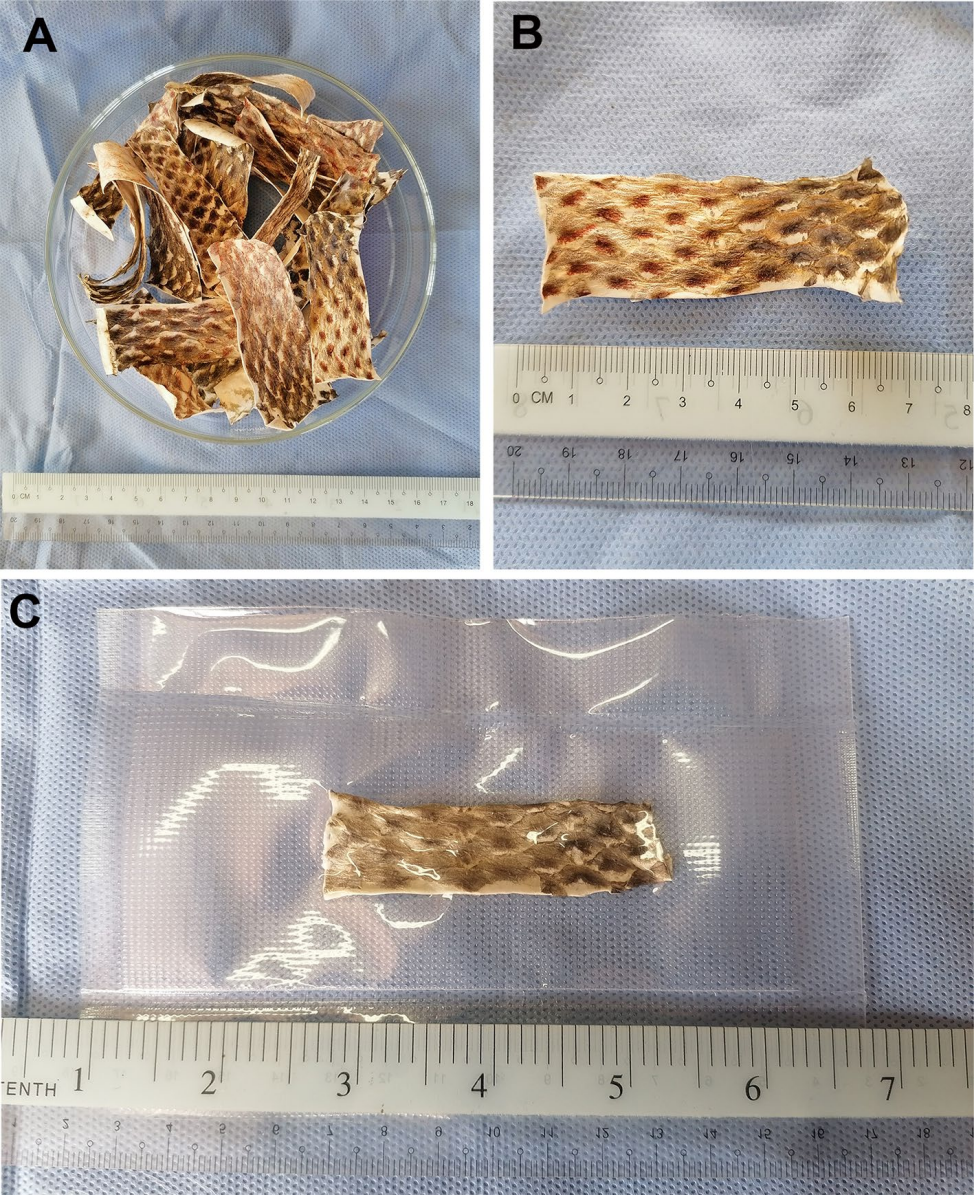
(**A**) and (**B**) Gross micrographs of the fish skin after the lyophilization, (**C**) vacuum- packaging.

Lyophilization was carried out in a freeze dryer (LYOQUEST, SPAIN, Serial No.: 1812, manufacture company: Telstar). Fresh fish skin strips were placed in the flasks of the freeze dryer apparatus at − 80 °C for 24 h. Skin strips were then subjected to a cold vacuum for 5–6 h. Lyophilized skin strips were then vacuum-packed (Fig. 1A–C).

### Gama (γ) irradiation sterilization of lyophilized fish skin

Gamma irradiation was performed at a gamma station (^60^Co gamma cell (2000 Ci), 30 ± 5 °C, 1.5 Gy/s, 150 rad/s), the National Center for Radiation Research and Technology (NCRRT) of the Egyptian Atomic Energy Authority, Cairo, Egypt. The vacuum-packed lyophilized Tilapia skin was subjected to Cobalt 60 γ sterilization at 5, 10, and 25 kGy^2^. Sterilized skin strips subjected to microbiological and histopathological evaluations after each treatment 15- and 30-days post-sterilization (3 skin strips each).

### Microbiological evaluation

Following Morton et al.^22^, the dilution plate method was employed to count the lyophilized fish skin microbiology under different lengths of gamma irradiation (0, 5, 10, and 25 KGy) after 15 and 30 days of irradiation. Fish skin strips were swabbed with a sterilized swab needle and suspended in 10 mL of sterile saline solution. For microbiological counting and identification, various media types were employed; nutrient agar and MacConkey agar media to count all aerobic bacteria, yeast malt extract medium (YME) to count the aerobic yeasts, and potato dextrose agar medium (PDA) to count the fungi^23–25^. One milliliter (1 mL) of the saline-swabbed solution was added to sterilized Petri dishes before the dishes were filled with sterilized isolation medium, three replicates were performed for each medium, and left to solidify. For bacteria, plates were incubated for 24 h at 35 °C, for yeasts, for 72 h at 28 °C, and for fungi, 7 days at 28 °C. The developed colony count was estimated as CFU/cm^2^. Using the same media, developed colonies with variations in their morphological characteristics, such as size, color, colony edge, and pigmentation, were sub-cultured and purified for identification using Bergey’s Manual of Systematic Bacteriology^26^, Yeasts: characteristics and identification^24^ and fungal identification^27^.

### Histological evaluation

Fish skin strips (0.5 × 0.5 cm) were fixed in 10% neutral buffered formalin, routinely processed, and subsequently embedded in paraffin. Afterwards, they were sectioned into 5 μm thick sections and stained with Mayer’s hematoxylin (Merck, Darmstadt, Germany) and eosin (Sigma, Missouri, USA). After microscopically examining the slides, histological evaluations were performed blindly on coded samples, with a comparison to control group. Collagen fibers integrity and organization were assessed histologically based on 0–3 scale^10,28,29^. Collagen fibers integrity scores: 0 = continue, long fiber, 1 = slightly fragmented, 2 = moderately fragmented, 3 = severely fragmented. Collagen fibers organization scores: 0 = compact and parallel, 1 = slightly loose and wave, 2 = moderately loose, wavy and cross to each other, 3 = no identifiable pattern.

### Histochemical evaluation

The collagen content evaluation was carried out by the Gomori’s trichrome stain^30^. After deparaffinization in xylene, paraffin-embedded sections were and rehydrated in a graded series of ethanol solutions (into 0.1 M phosphate-buffered saline (PBS), pH 7.2) to distilled water. Sections were stained with Gomori’s trichrome according to the manufacturer’s protocol, dehydrated in graded alcohol, made transparent with xylene, and mounted. Slides were then microscopically examined to verify collagen staining with green. The collagen content was evaluated based on the depth of the green staining.

The percentage of collagen-positive area was calculated by ImageJ (1.48v) using threshold area fraction determination. The amount of collagen was expressed as a percentage from the total number of pixels in the optical view as a percentage and expressed as mean ± SEM.

### Statistical analysis

One-way ANOVA followed by Tukey's post hoc test was performed using GraphPad Prism software version 8.0.1 (GraphPad Software Inc., La Jolla, CA, USA). *P* values < 0.05 were considered statistically significant.

## Results

### Microbiological evaluation of the lyophilized fish skin after gamma irradiation

**Figure 2:**
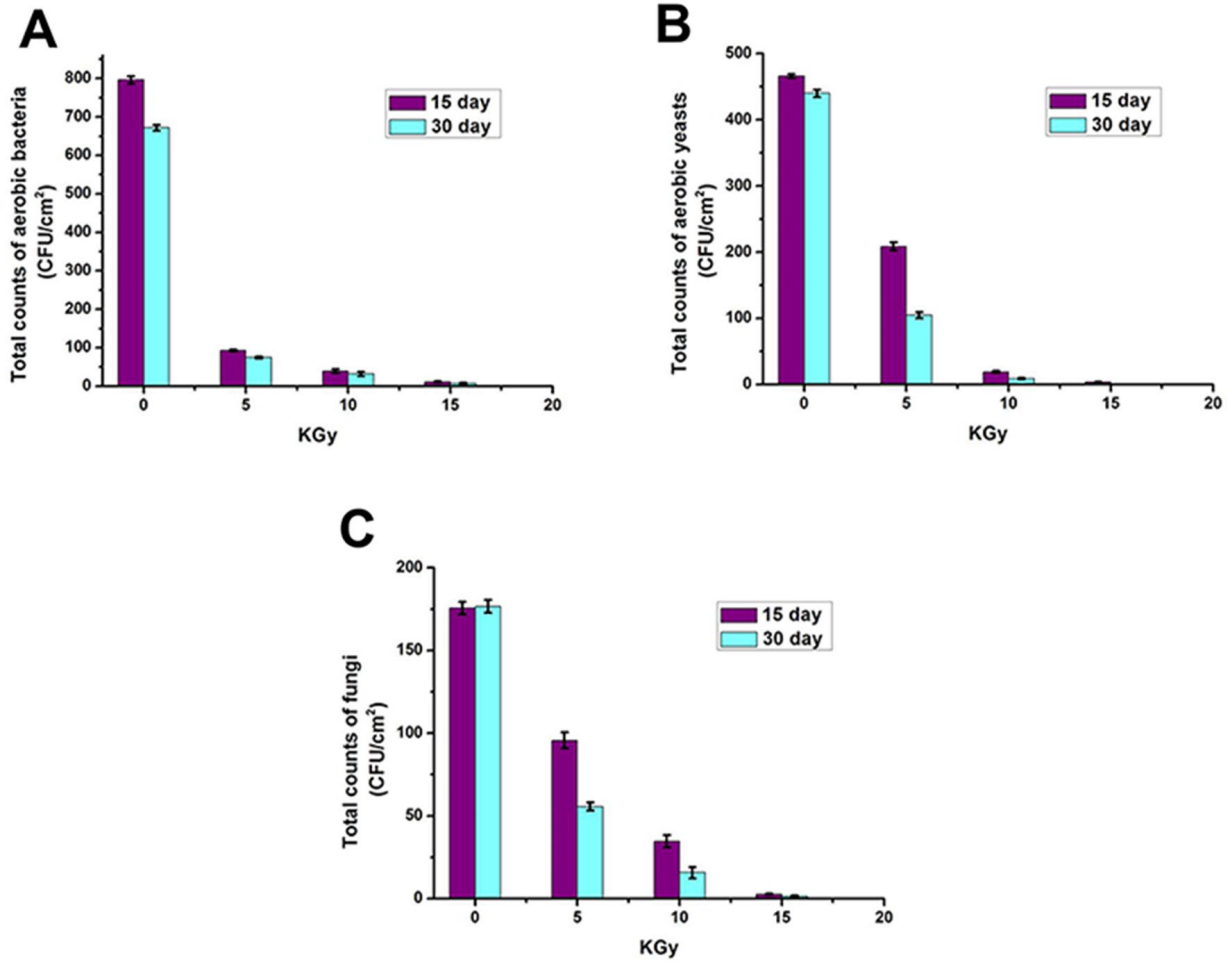
Total accounts (CFU/cm^2^ ± S.D) of aerobic bacteria (**A**), aerobic yeasts (**B**), and aerobic filamentous fungi (**A**) on lyophilized fish skin sterilized with gamma irradiation (0, 5, 10, 25).

Gamma irradiation exhibited an efficient sterilizing effect on fish skin surface microbiota. Different lengths of gamma irradiation (5, 10, and 25 KGy) were applied to the lyophilized fish skin. The microbial counts of aerobic bacteria, aerobic yeasts, and fungi were detected 15- and 30- days after the irradiation as cleared in Fig. 2A–C. It was clear that gamma irradiation is a great microbial sterilizer, especially with the high length of 25 KGy that inhibited the aerobic bacterial counts significantly by 98.53% and 98.96%, aerobic yeast counts by 99.2% and 99.8%, and fungal counts by 98.48% and 99.25% 15- and 30- days after irradiation, respectively, (*P* < 0.05). Gamma irradiation also maintained the low microbial skin counts for a longer period (30 days) and remarkably at the low length of 5 KGy, gamma irradiation was effective against aerobic bacteria.

By increasing the gamma irradiation, the total counts of aerobic bacteria decrease dramatically giving 92.67 ± 2.62 (88.36% inhibition), 39.33 ± 4.92 (95.06% inhibition), and 11.67 ± 1.7 (98.53% inhibition) at 5, 10, and 25 KGy 15 days post-irradiation, respectively, (*P* < 0.05). At 30 days post-irradiation, the bacterial counts were 74.33 ± 2.49 (88.93% inhibition), 32 ± 5.7 (95.24% inhibition), and 7 ± 1.6 (98.96% inhibition) at 5, 10, and 25 KGy, respectively, compared to the untreated sample (796 ± 9.93 and 671.7 ± 7.4) 15- and 30-days after irradiation, respectively, (*P* < 0.05).

For yeasts, it was clear that by increasing the length of gamma irradiation, the total counts of yeasts decreased significantly after 15- and 30-days irradiation giving 208.67 ± 6.13 (55.25% inhibition), 19 ± 1.63 (95.93% inhibition), and 3.67 ± 0.47 (99.2% inhibition) at 5, 10, and 25 KGy, respectively, at 15 days (*P* < 0.05). At 30 days, the yeast counts were 104.67 ± 4.9 (76.21% inhibition), 8.7 ± 1.2 (98.03% inhibition), and 1 ± 0 (99.77% inhibition) at 5, 10, and 25 KGy, respectively, compared to the untreated sample (466.3 ± 2.87 and 440 ± 5.72) 15- and 30-days after irradiation, respectively, (*P* < 0.05).

Filamentous fungi had the same criteria as bacteria and yeasts, the total counts of fungi decreased significantly (*P* < 0.05) with increasing the length of gamma irradiation giving 95.7 ± 4.9 (45.54% inhibition), 34.7 ± 3.7 (80.27% inhibition), and 2.67 ± 0.4, (98.48% inhibition) at 5, 10, and 25 KGy, respectively, after 15 days. After 30 days, the yeast counts were 55.67 ± 4.9 (68.49% inhibition), 15.7 ± 3.3 (91.13% inhibition), and 1.3 ± 0.1 (99.24% inhibition) at 5, 10, and 25 KGy, respectively, compared to the untreated sample (175.7 ± 3.7 and 176.67 ± 3.8) 15- and 30-days after irradiation, respectively.

By investigating the microbial species present on the fish skin, *Bacillus* sp., *Escherichia coli, Micrococcus luteus,* and *Serratia marcescens* were the dominant aerobic bacteria, *Candida* sp., *Saccharomyces* sp. and *Rhodotorula* sp. were the dominant aerobic yeasts, whereas *Aspergillus niger, A. flavus, A. fumigatus,* and *Rhizopus stolonifer* were the dominant aerobic fungi**.**

### Impact of lyophilization on fish skin

**Figure 3:**
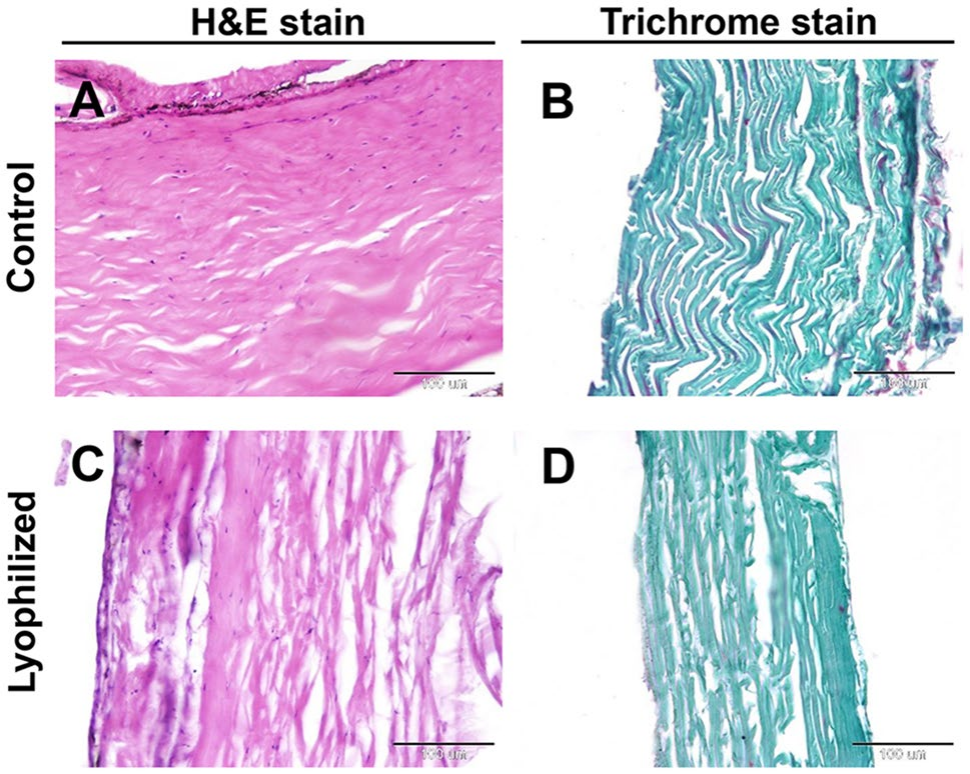
(**A**) and (**B**) Histological and histochemical evaluations of the fresh Tilapia skin, (**C**) and (D) the lyophilized Tilapia skin. (**A**, **C**) Hematoxylin and eosin-stained sections. (**B**, **D**) Gomori’s trichrome-stained sections for collagen. The scale bars = 100 μm.

In the control group, fresh fish skin was examined histologically, and the collagen fibers were tightly packed, well-organized, parallel-distributed, and no evidence of disaggregation (Fig. 3A,B). In addition, the lyophilized fish skin rehydrated in normal saline for 15 min showed the preservation of the collagen fibers in a well-organized pattern with no signs of disaggregation (Fig. 3C,D).

### Histological evaluation of the lyophilized fish skin after gamma irradiation

**Figure 4:**
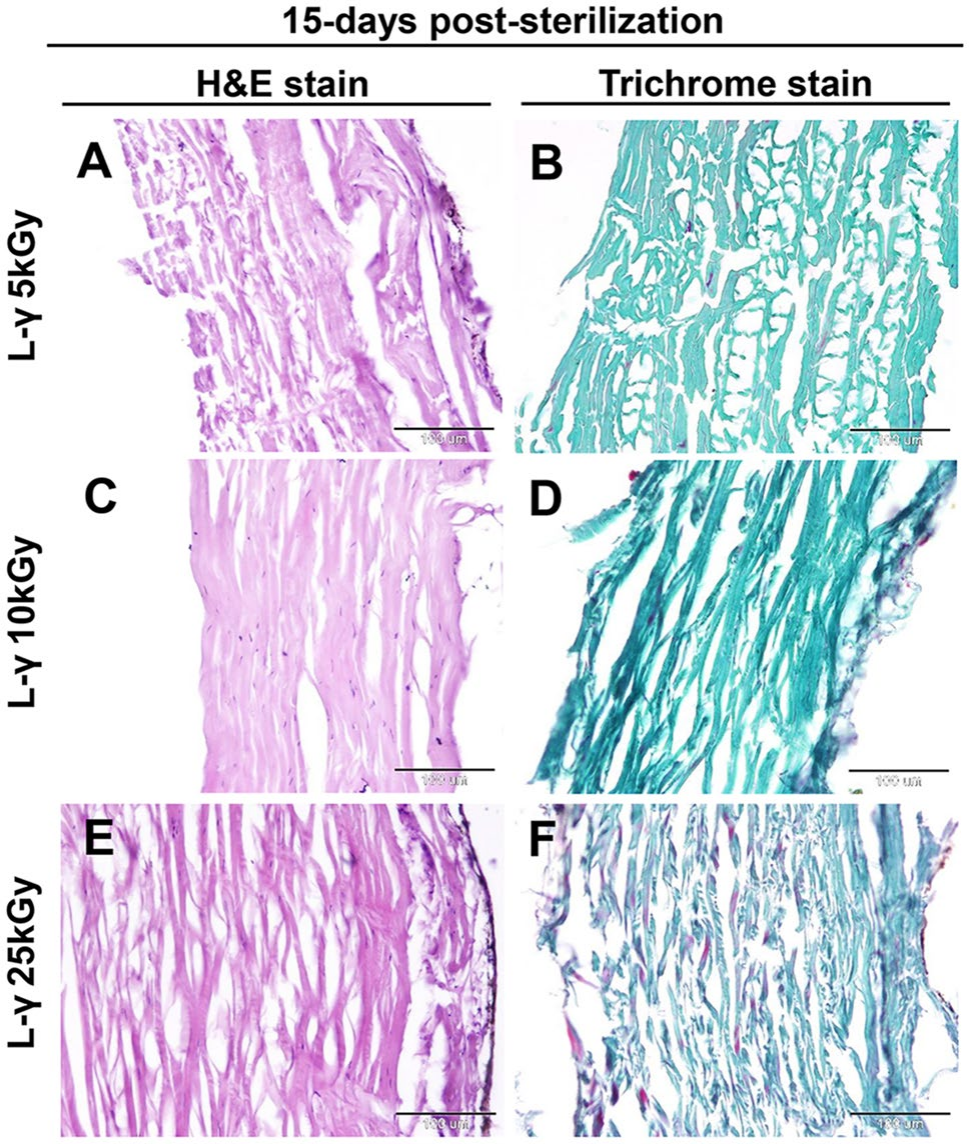
Histological and histochemical evaluations of the lyophilized Tilapia fish skin submitted to different irradiation dosages 15-days post-sterilization. Hematoxylin and eosin stained sections were irradiated at 5 (**A**), 10 (**C**), and 25 (**E**) kGy. (**B**), (**D**), and (**F**) Gomori’s trichrome stained sections for collagen. The scale bars = 100 μm.

**Figure 5:**
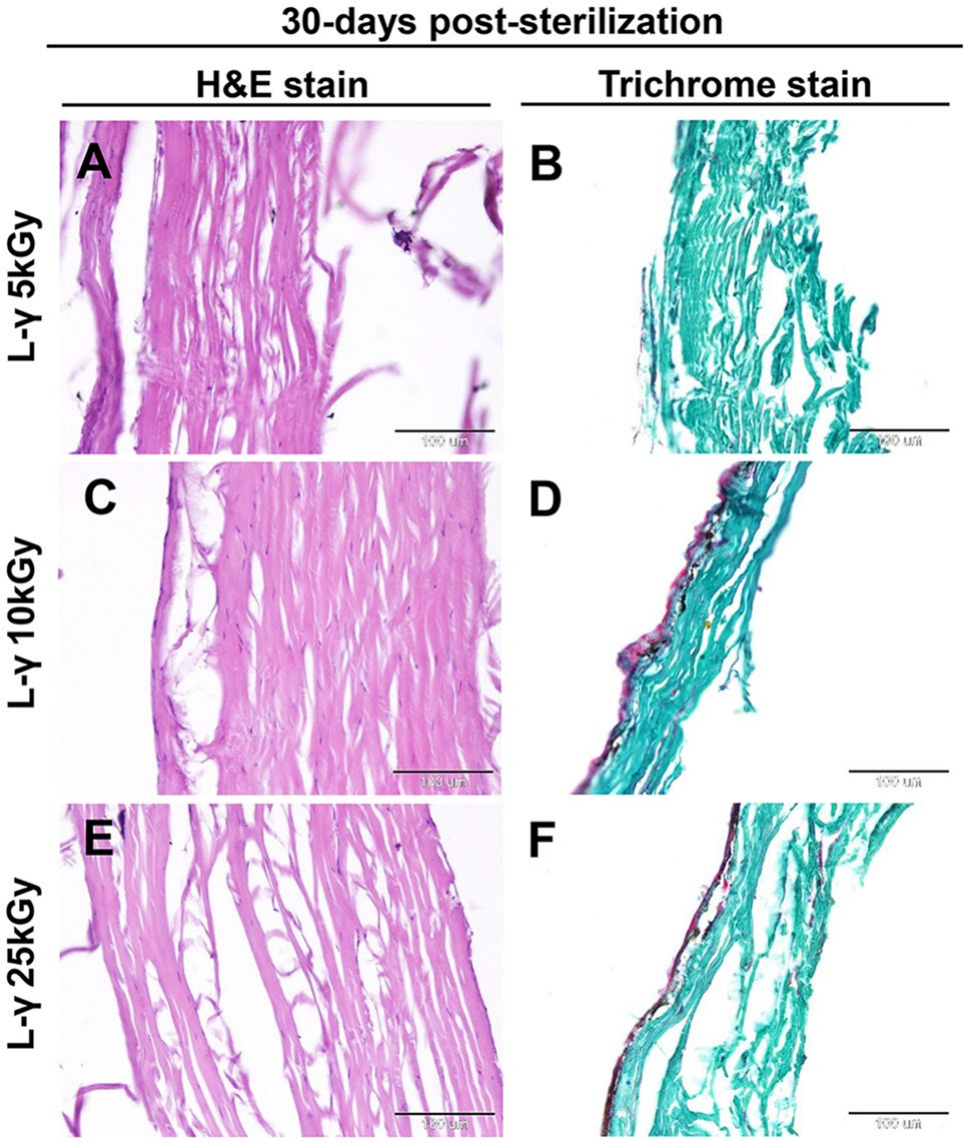
Histological and histochemical evaluations of the lyophilized Tilapia fish skin submitted to different irradiation dosages 30-days post-sterilization. Hematoxylin and eosin-stained sections were irradiated at 5 (**A**), 10 (**C**), and 25 (**E**) kGy. (**B**), (**D**), and (**F**) Gomori’s trichrome stained sections for collagen. The scale bars = 100 μm.

**Figure 6:**
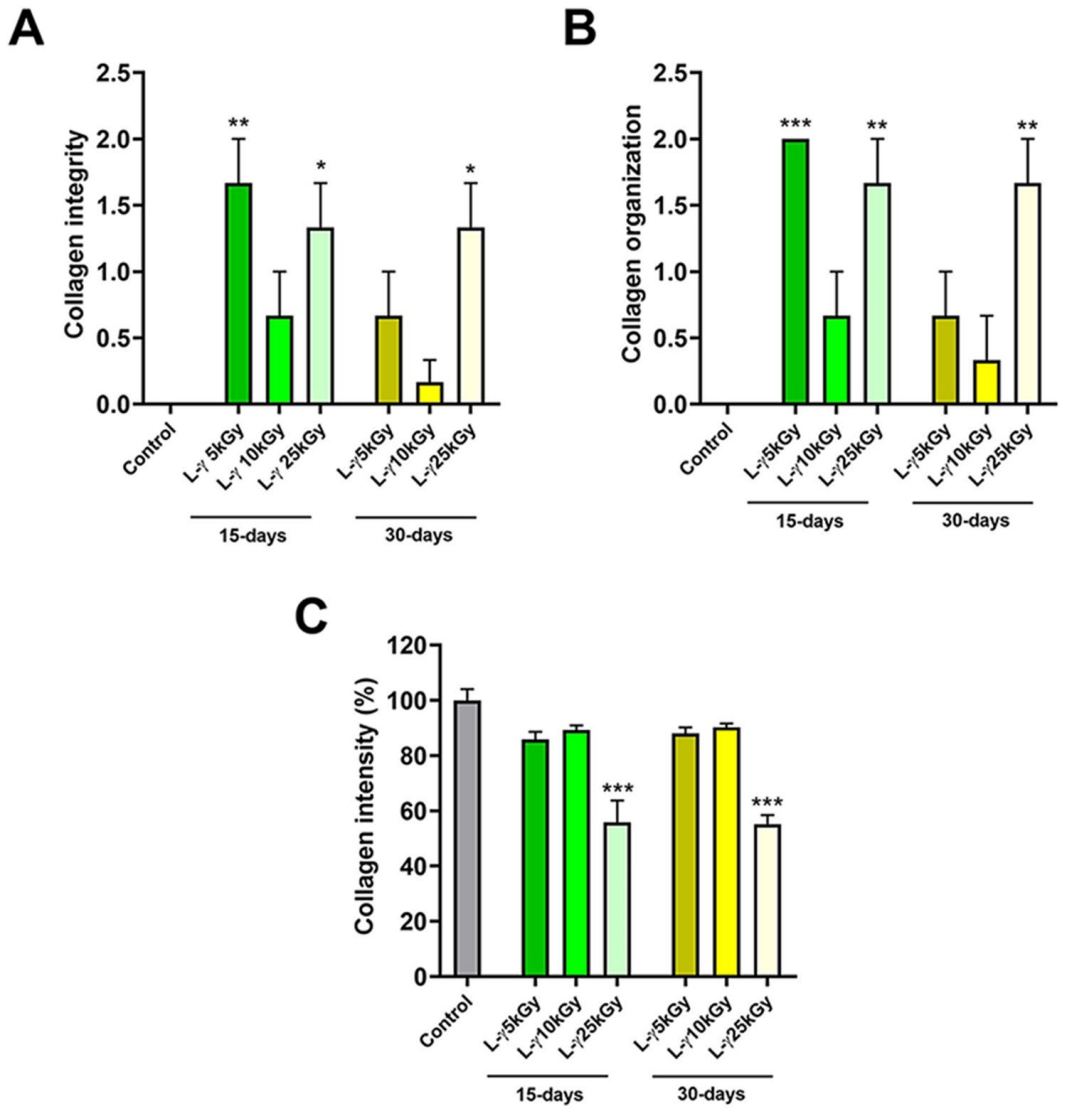
Evaluation of the collagen integrity, organization, and intensity in the lyophilized Tilapia fish skin submitted to different irradiation dosages. Collagen integrity and organization were evaluated based on a 0–3 scale as described in the materials and methods. The collagen intensity was quantified using ImageJ. The results are expressed as a percentage of the total number of pixels and are normalized to the control-treated group. Differences were evaluated using one*-*way ANOVA. *p < 0.05; **p < 0.001; ***p < 0.0001.

In the lyophilized fish skin subjected to gamma irradiation at 5, 10, and 25 kGy, histological examination of the fish skin 15-day post-sterilization revealed slightly to moderately disorganized and disaggregated in irradiated skin at 5 and 25 kGy (Figs. 4A,E, 6A,B) (*P* < 0.05). However, the collagen fibers were well organized in a parallel pattern at 10 kGy (Fig. 4C). At 30-days post-sterilization, the collagen fiber structure did not change in the irradiated skin at 5 and 10 kGy (Figs. 5A,C, 6A,B) (*P* < 0.05), with disorganization and disaggregation were more prominent at 25 kGy (Figs. 5E, 6A,B) (*P* < 0.05).

### Histochemical evaluation of the fish skin

The collagen fibers were also stained with Gomori’s trichrome stain and the collagen intensity in the skin was measured using ImageJ (1.48v). At 15- and 30-days post-sterilization, the collagen disposition and intensity in skin samples irradiated at 5 and 10 kGy did not show significant differences compared with the control (Figs. 4B,D, 5B,D, 6C). However, collagen deposition and intensity were significantly decreased in skin samples exposed to 25 kGy after 15 and 30 days of irradiation (Figs. 4F, 5F, 6C) (*P* < 0.0001).

## Discussion

Recently, biological dressings have become indispensable in modern strategies of burn and wound management. The current study investigated the use of lyophilization as a method for the long-term preservation of fish skin grafts in a manner that maintains the integrity of fish skin collagen. Moreover, the study revealed that gamma irradiation at a length of 10 KGy is optimal for sterilizing the lyophilized fish skin grafts without disrupting the collagen content.

Autologous skin grafts have a limited availability and add an additional scaring^31^. Allografts have many limitations such as the finding of suitable donors and the risk of infection transmission, especially, viral infections^20^. Therefore, various xenografts have been described as biological wound dressings in humans. This includes the bovine embryo skin^32^, bovine amnion^33^, canine skin^34^, frog skin^35^, and porcine skin^36–38^. However, there are concerns about their clinical application due to immunological causes, zoonotic risk, and religious beliefs^20,39,40^.

The fish skin was found to be consistent with human skin^8,13^ and has a higher collagen content than other skins^9,13,14^, which is a potential wound healing promotor^41^. Moreover, fish skin has advantages such as low antigenicity, a high potential adherent to the skin, a lower risk of transmitting diseases, and a degree of moisture symbolizing the human skin^8,42^. Fish skin graft is highly porous. Since the pore size is within the range of typical cell size, fish skin is well suited to support cell ingrowth^43^. Therefore, the fish skin has recently been suggested as a potential xenograft^1–7,9–12^.

Although the tilapia skin contains a normal non-infectious microbiota, it may be exposed to microbial contaminants from a contaminated water environment^7,44^. Bacteria, yeasts, fungi, and viruses represent serious fish contaminants^45^. Enterobacteriaceae, yeast, and aerobic spoilers are the main spoilage microorganisms of fish during the storage^46^. Due to the high-water activity, and low acidity in the fish skin, bacteria proliferate quickly causing its spoilage^47^. Therefore, in most studies, Tilapia skin was used as a biological dressing graft for burn or wound treatment shortly after its processing^1,3–7,9,10^. This creates an urgent need to find a way for long-term preservation and storage of the fish skin grafts.

Various methods have been used for the preservation of different biological dressings, such as cryopreservation^48,49^ and glycerolization^4,5,7,9,50–52^. Although these methods could prolong the shelf life of biological dressings, they may also affect their effectiveness^53^.

Lyophilization or freeze-drying is a specific dehydration process, which provides benefits to the final product through improved composition stability and reduced water content to low levels, which allow the cell to survive during long storage^18,54^. Microbial cells need high stabilization processes to survive on the lyophilized parts. Otherwise, they will be damaged and/or die^55^.

Lyophilization has been addressed in previous studies for fish skin preservation^2,3^. However, using a rigorous process of chemical sterilization of the fish skin before the lyophilization raises concerns about collagen integrity and other biological components potential for healing^43^, especially, since these studies were not followed by histological evaluation before clinical application^1,2,7^. Moreover, using several sterilizing steps with several chemicals make such procedures cost-effective and impractical for industrial application. Chlorhexidine is commonly used to sterilize and disinfect grafting procedures. However, many bacterial spores or mycobacteria are chlorhexidine-resistant^56,57^. It also has a low activity against viruses. A potential limitation of chlorhexidine is its cytotoxicity and alteration in the biochemical properties of collagen^10,58^.

Here, this study describes the lyophilization of fish skin without using any disinfectants during the processing of fish skin grafts to ensure the collagen safety and subsequently its effectiveness. This was confirmed by histological and histochemical evaluations of the lyophilized fish skin after rehydration in normal saline for 15 min that there was no change in the arrangement and content of the collagen fibers of the fish skin.

Sterilization of fish skin with gamma irradiation represents an efficient sterilizing method for the fish skin microbiota. High-energy gamma irradiations are released by several radioisotopes, including the comparatively cheap byproducts of nuclear fission Caesium-137 (137Cs) and Cobalt-60 (60Co). By subjecting the plentiful, non-radioactive Co-59 isotope to neutron irradiation inside a nuclear reactor, radioactive Co is created. Then, after emitting one electron and two gamma rays with energies of 1.17 MeV and 1.33 MeV, Co-60 atoms decompose into nonradioactive Ni-60 atoms. Gamma rays don't have enough energy to cause radioactivity in other materials because they are released isotropically. Similar to x-rays, gamma rays have a shorter wavelength and higher energy. Thus, Gamma rays are appealing for industrial sterilization of materials with a significant thickness or volume, such as packaged food, medical equipment, or medical supplies^59^.

The combination of lyophilization and gamma irradiation inhibited the microbial growth by 99.8%. Gram-negative and Gram-positive bacteria, as proteobacteria and the Lactobacillales, are more vulnerable to Gamma irradiation than spore-forming bacteria, such as Bacilli and Clostridia^60^. This is in accordance with our findings that *Bacillus* sp., *Escherichia coli, Micrococcus luteus,* and *Serratia marcescens* were the dominant aerobic bacteria. Similar findings were recorded by Dharmarha et al.^61^, who found that Gamma irradiation decreased *Pseudomonas* sp., *Escherichia coli*, and Yersinia sp. total counts highly than spore-forming bacteria. Gamma irradiation was found to have an effect on the microbial DNA by altering its composition^21^. It reacts with water molecules forming free radicals damage DNA and breakage the DNA double strand with non-repaired effects causing microbial cell death^62^.

An infection is deemed to exist when there are more than 10^5^ CFU of bacteria per gram of skin graft^63^. On wound beds with more than 10^5^ bacteria/g of tissue, the absorption of a skin graft is decreased^64^. In our results, the total bacterial counts were less than 35 CFU/cm^2^ at 10 and 25 kGy after 30 days of sterilization, indicating the high sterilizing efficiency of these irradiations; however, 10 kGy is preferred as it maintains the collagen integrity and content. Also, the total counts of yeasts and fungi were less than 10 and 16 CFU/cm^2^, respectively, at 10 and 25 kGy after 30 days. In accordance with our results, clinical investigations found that skin transplant failure occurred in wounds that were significantly infected with 10^7^ pathogens^65,66^. Also, Nsaful et al.^67^ found that wound graft failure mainly occurs by the microbial contamination, especially bacterial contamination with the bacteria counts ranged from 3.7 × 10^5^ to 9 × 10^5^ CFU/cm^2^.

In accordance, histological evaluation of the fish skin grafts revealed that the exposure to gamma irradiation at 25 kGy caused the collagen fibers to dissociate and disintegrate with a reduction in the collagen intensity, whereas exposure at 5 kGy altered collagen fiber arrangement and integrity. However, the exposure to gamma irradiation at 10 kGy showed the preferred intensity since it preserved the collagen fiber content and intensity in the skin graft.

The limitation of this study is the lack of histological and microbiological assessment of the sterilized vacuum-packed lyophilized fish skin grafts on further long period (6 and 12 months). Therefore, future studies should be conducted to address this concern.

## Conclusion

This study established an optimized method for tilapia skin lyophilization. It defined the standard length of gamma irradiation for sterilizing lyophilized tilapia skin, considering the collagen integrity and microbial count of fish skin. These processed, sterilized, vacuum-packed, lyophilized fish skin patches could be suitable for long-term storage for burn and wound management in hospitals and medical centers. Further clinical studies are still being conducted on this product.

## Data Availability

All data generated or analyzed during this study are included in this published article.
